# The mobile vaccine equity enhancement program–a model program for enhancing equity in vaccine availability based at a large health care system

**DOI:** 10.3389/fpubh.2023.1271162

**Published:** 2023-10-17

**Authors:** John Broach, Olga Brown, Caitlin McEachern, Janell Forget, Peter Lancette, Norman Soucie, Julie Inzerillo, Robert Klugman, Stephen Tosi, Abraham Haddad, Pamela Manor, Richard Bylund, Gio Dellostritto, Max Grecchi, Connie Camelo, Jeanne Shirshac, Katharine Eshghi, Nardy Vega, Stacy Hampson, Kassandra Follwell, Rafael Gonzalez, Theresa Hicks, Victoria McCandless, Timothy VanStratten, Mina Botros, Tracy Jalbert, Catherine Merwin, Wendy Schellhammer, Ian Pelto, Maggie Rodriguez, Cheryl LaPriore, Monica Lowell, Elizabeth Radigan, Lorie Gull, Alana Gruszecki, Sarah Benoit, Eric Dickson, Michelle Muller

**Affiliations:** ^1^University of Massachusetts Medical School, Worcester, MA, United States; ^2^UMass Memorial Medical Center, Worcester, MA, United States; ^3^UMass Memorial Health Care, Worcester, MA, United States; ^4^Fairlawn Rehabilitation Hospital, Worcester, MA, United States; ^5^University of Colorado Anschutz Medical Campus, Aurora, CO, United States

**Keywords:** vaccine, COVID, pandemic (COVID19), equity, mobile healthcare application

## Abstract

The SARS CoV-2 (COVID-19) pandemic presented unprecedented challenges as communities attempted to respond to the administration of a novel vaccine that faced cold chain logistical requirements and vaccine hesitancy among many, as well as complicated phased rollout plans that changed frequently as availability of the vaccine waxed and waned. The COVID-19 pandemic also disproportionately affected communities of color and communities with barriers to accessing healthcare. In the setting of these difficulties, a program was created specifically to address inequity in vaccine administration with a focus on communities of color and linguistic diversity as well as those who had technological barriers to online sign-up processes common at mass vaccination sites. This effort, the Mobile Vaccine Equity Enhancement Program (MVeeP), delivered over 12,000 vaccines in 24 months through a reproducible set of practices that can inform equity-driven vaccine efforts in future pandemics.

## Introduction

As of March of 2023, the COVID-19 pandemic caused over 676 million documented cases worldwide resulting in at least 6,881,000 deaths (1). In the US alone, there have been over 100 million cases of COVID-19 and, prior to the availability of the first COVID-19 vaccine in late 2020, over 800 thousand Americans had died from the infection (2–4). Due to the incredible burden of this disease, and its disproportionate impact on communities of color, older populations, and those with limited English proficiency, it was apparent that once a life-saving vaccine was available, it would be important to ensure that it was made available in an equitable fashion. Mass vaccination sites were a critical component of early vaccine distribution, especially as states tried to achieve vaccination rates as high as possible as early as possible in 2021 (5, 6). While these sites were efficient at providing large numbers of vaccines, many people found it challenging to use the internet-based scheduling system, to find appointments, and to find transportation to those appointments. Indeed, some individuals at greatest risk, such as the older adult and homebound, were completely unable to access vaccines (7–9). Beyond technological challenges, equity in vaccine distribution suffered as well, partially related to mass vaccination sites favoring those with access to transportation, those who lived near the vaccination centers, and those without socioeconomic barriers to seeking care at these facilities (10–14). While Massachusetts was successful in vaccinating a large percentage of its population, a need clearly existed to extend vaccine capability to those with difficulty accessing mass vaccination sites and to ensure equitable distribution of this life-saving measure.

Tertiary care medical centers are uniquely positioned with medical assets and community connections and were partners in numerous COVID-19 mitigation efforts. The vaccine effort described here, known as the Mobile Vaccine Equity Enhancement Program (MVeeP), was a program created by one such institution, UMass Memorial Health, Inc., in order to enhance the equitable availability of the COVID-19 vaccine in its community.

## Context

After the first COVID-19 vaccine became available in December of 2020, work began to create a mobile and community-focused vaccine effort with the capability to provide vaccinations in community settings and in the homes of patients who could not easily access other sites. The concept of operations for our program was a mobile service, capable of administering vaccines at community sites or in the homes of individuals, and which focused on accessing individuals with barriers to other sites. Several factors were considered when determining the best sites to target for vaccination clinics including the need to make the service available to individuals who did not speak English as a primary language, had barriers to transportation, were at particularly high risk for severe COVID-19 infection, and communities of color. In particular, at the time of this intervention and since, African American and Latinx individuals were underrepresented among the vaccinated population, and particular attention is required to ensure that vaccination is made available in an equitable manner (15). It was also recognized that a number of high-risk individuals with advanced age and multiple comorbidities might have difficulty accessing the standard online sign-up portals for mass vaccination sites and, indeed, were more likely to be home-bound or to have challenges with reliable transportation.

The MVeeP effort was undertaken in the larger context of a health-system-wide approach to ensure vaccination for the patients in our catchment area of Central Massachusetts. This meant that this mobile, equity-focused intervention was coupled with a strategy to vaccinate caregivers, provide vaccinations for existing patients who could attend their in-office PCP appointments or present to one of the system hospitals for vaccination or receive it as part of an Emergency Department or hospital stay. Several of the specific considerations for vaccine roll-out and site selection, along with the response posture adopted by MVeeP are described below.

### Phased vaccine roll out

Throughout this effort, several factors influenced target populations and the operational plan including: vaccine availability, community engagement and outreach, administration reporting requirements, administration regulations, and storage and logistics.

The State of Massachusetts proceeded during early vaccine distribution with a phased rollout of eligibility similar to other states (Table 1). All vaccine providers were required to comply with this eligibility schedule and to attest to the fact that the individuals being vaccinated were appropriate at the time that their dose was being given. As the MVeeP program selected sites and engaged with community leaders, these eligibility criteria were strictly adhered to.

**Table 1:** Initial COVID-19 Vaccine Phased Rollout Schedule – Massachusetts Department of Public Health.

Phase	Date	Group
1	12/15/20	Clinical and non-clinical healthcare workers doing direct and COVID-facing care
	12/28/20	Long-term care facilities, rest homes, and assisted living facilities
	1/11/21	First responders
	1/18/21	Congregate care settings
	1/21/21	Home-based healthcare workers Healthcare workers doing non-COVID-facing care
2	2/1/21	People who are 75 or older
	2/18/21	People who are 65 or older People with 2 or more certain medical conditions People who live or work in low-income and affordable senior housing
	3/11/21	K-12 educators, K-12 school staff, and childcare workers
	3/22/21	People who are 60 or older Workers in certain categories
	4/5/21	People who are 55 or older People with 1 or more certain medical conditions
3	4/19/21	People age 16 and older who live, work or study in Massachusetts
	5/12/21	People age 12 and older who live, work or study in Massachusetts
4	11/3/21	Children age 5–11 who live or study in Massachusetts
5	6/20/22	Children ages 6 months to 4 years old who live or study in Massachusetts

https://www.mass.gov/info-details/massachusetts-covid-19-vaccination-phases#phase-1-

In the State of Massachusetts, registered vaccine administrations are tracked according to an organizational personal identification number (PIN) and logged into the Massachusetts Immunization Information System (MIIS). Each dose is ordered and delivered to the PIN holder and that entity is responsible for ensuring that each vaccine administration is accurately entered into the online system. Requirements for PIN holders include verification of the ability to receive and store vaccines, as well as an authorized ordering provider. Early in the vaccine effort, it was clear that health centers would play a role in distribution but it was unclear how large a role or how many doses they would be allotted. Numerous locales used a wide variety of vaccine deployment strategies in the U.S. These included partnerships with health systems, programs managed by municipalities, towns, counties, etc. and others focused primarily on state-sponsored programs (16–18). Our program was able to use the PIN associated with the UMass Memorial Medical Center; its pharmacy ordered vaccines based on perceived demand across a number of vaccine efforts including this program, inpatient vaccination efforts, employee vaccination efforts, and large scale vaccination efforts.

## Detail to understand key programmatic elements

### Vaccine scarcity management

In the early phases of vaccine administration, drug supply and allocation played a significant role in guiding prioritization efforts. In addition, vaccine scarcity meant that any vaccine-administering organization in Massachusetts was held accountable for each dose given and were entrusted to ensure that no doses were wasted. Complicating this further was the fact that each vaccine vial had a predicted number of doses contained within, yet the vials were frequently found to have a small amount of overfill that allowed some providers to obtain an extra dose from some vials if the medication was drawn up carefully.

### Vaccine draw logistics and scheduling

For events at which hundreds or thousands of recipients were expected, these overages and accidental wastage could be balanced over the course of multiple vials. However, for small, targeted community events at which individuals with difficult access were targeted, this reality meant that a sophisticated system was needed to ensure that each dose was allotted and that no doses were wasted. Adding to the challenge was the fact that, once removed from storage, each dose was only usable for 2 h, meaning that not all vaccines could be drawn up at the beginning of the event in order to have a single starting count since it was common to have extra doses obtained as the vaccines were being drawn from the vials. It was also the case that, as numerous cases of COVID-19 were occurring during the vaccine administration timeframe, it was not uncommon to have patients signed up for the vaccine, who then had to withdraw. This created a situation where the MVeeP team had to plan on a certain number of vaccines being available, overbook the event to some extent based on local prevalence, and also have a roster of “stand-by” individuals available to quickly come to the site or who lived within a certain radius to ensure that extra doses if available, could be administered in the allotted time frame after they were drawn out of the vial. Key to this system was the role of the *Vaccine Navigator*. This role was a critical intervention that contributed to the success of the effort and is discussed in more detail below.

### Vaccine scheduling and walk-ins

The purpose of this program was to accommodate individuals who had barriers to or challenges with accessing an existing mass vaccination site. For this reason, MVeeP clinics were run with partner agencies at various locations in the community. The program evolved as vaccines became more available to accommodate walk-in participants but, early on, visits were scheduled ahead of time so that vaccine availability could be assured. In order to schedule visits, we worked closely with our community partners to ensure that the events were scheduled at times that made it easy for people to access them (i.e., change of shift at employer-based clinics to accommodate both off-going and oncoming workers). In addition, each partner helped provide a list of eligible individuals ahead of time. A customized Epic EHR software module was used to document the clinical process from scheduling appointments to the completion of vaccination. Epic EHR software also permitted on-site registration to accommodate walk-in patients and stand-by patients who were called to fill in when extra doses of vaccine were available.

### Vaccine navigator

The *Vaccine Navigator* role was established to ensure that the MVeeP program both had success in accessing communities that were underrepresented in vaccine administration as well as developing, maintaining and utilizing a list of “stand-by” patients. The Navigator was engaged in both the initial community outreach as the vaccine events were being organized and then was present for the entire vaccine event. During the event these individuals monitored the vaccine distribution, contacted patients that did not show up as planned, and when it was likely that extra vaccine doses would be available, contacted stand-by patients. Stand-by patients still needed to meet criteria for vaccine administration in a given phase and a specific point in time in the vaccine phase timeline. A running count of vaccine availability and the number of remaining scheduled patients were closely monitored to ensure that no dose was left ungiven, and no scheduled patient was denied a vaccine.

### Vaccine administration

**Figure 1:**
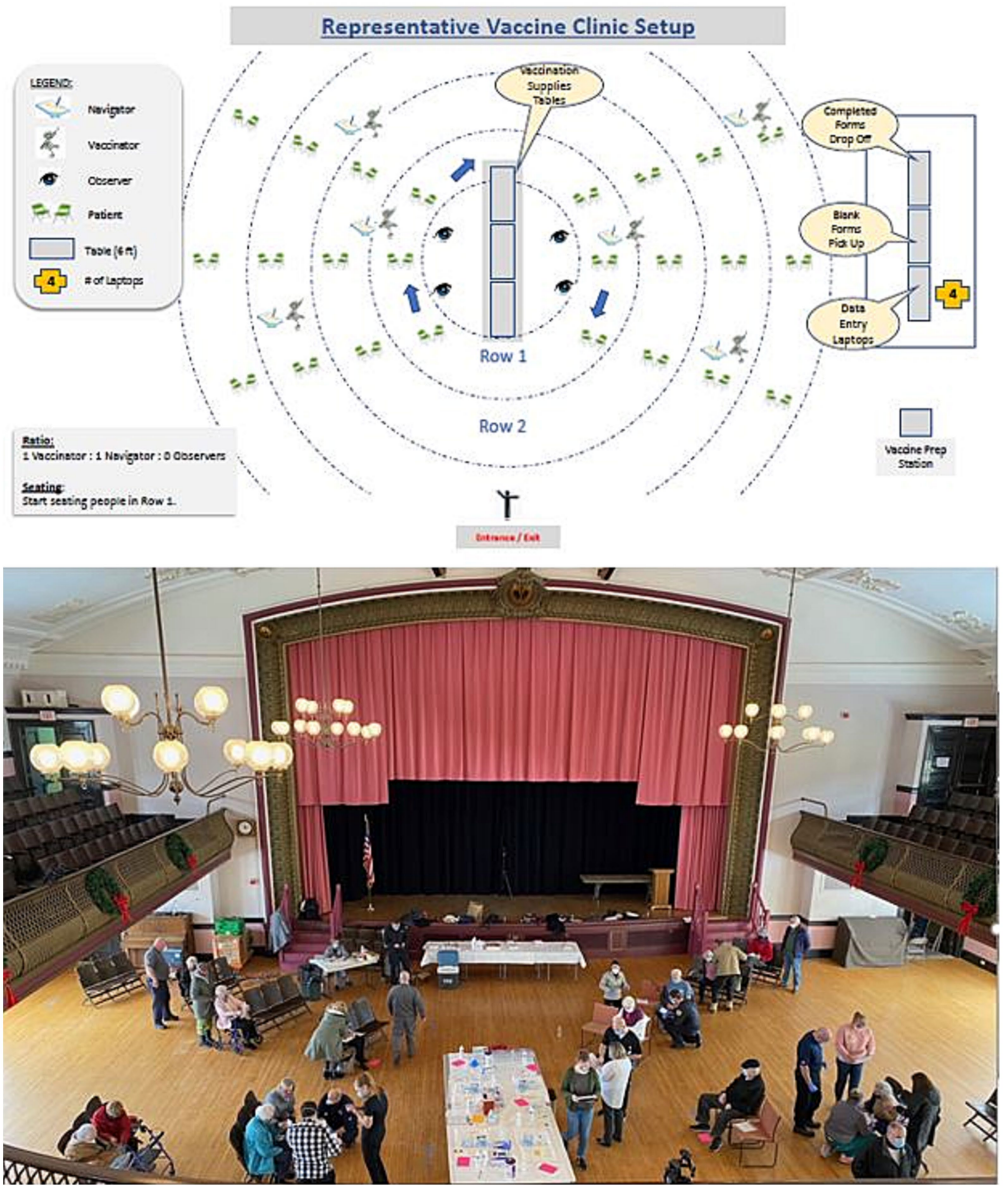
Typical MVeeP event set up schematic with representative photo of an event held in Clinton, MA.

On each vaccine clinic day, both administrative and clinical staff and volunteers were present. In general, registration and other administrative staff ensured that all patients were checked in, that they had all information correctly documented in the EHR, and that they had any questions answered. They were also supplied with information about the vaccine so that they had an opportunity to review it and ask questions of the clinical staff. Unlike other mass vaccination efforts where patients walked through various stations to have different parts of the check in, information dissemination, vaccine administration, and observation period performed, our program recognized that this would likely pose a challenge to mobility impaired individuals. For this reason, individuals were seated after check-in and all subsequent steps were completed in their seat. Documentation was completed in a mobile fashion using smart devices on which the Rover application had been installed. Once the vaccine was administered, the patient was given a piece of colored paper with the time at which their observation period would end. Staff were moving continuously throughout the area to monitor these times and to be alert for vaccine reactions. When it was noted that a patient had completed the observation period they were guided to the exit with assistance as needed and the surfaces of their seat were cleaned by staff prior to the next patient. In this way, efficient flow of patients could be maintained through the vaccination site. Figure 1 represents a schematic view and photographic representation of the important components of the mobile vaccine.

### Vaccines administered

The MVeeP had its first vaccination clinic on February 5, 2021 and concluded its operation on January 30, 2023. During these 24 months of operation, the program administered 12,117 vaccine doses to 8,545 unique individuals who received at least one dose of a two-dose vaccine (Moderna or Pfizer) or the single dose Janssen vaccination. We found that a significant percentage of patients had more than one vaccine administered by MVeeP with 36% receiving at least two doses and 5% receiving three or more. In this program, single dose vaccines were offered as well as initial two dose series, monovalent boosters, and bivalent boosters depending upon CDC and State of Massachusetts DPH recommendations at the time of administration.

In-home vaccinations were provided to 593 individuals who received a total of 748 vaccine doses. The 12,117 doses administered by MVeeP accounted for 11.9% of the total vaccines administered by the UMass Memorial Health system as of March, 2023. Data was pulled from the electronic health record and checked against both encounters for vaccination and actual administration of vaccination.

A total of 302 vaccine events were held with an average number of encounters at each event being 40 and the largest event providing vaccination to 328 patients in a single day.

An overview of the vaccine program is presented as Table 2.

**Table 2:** MVeeP vaccine encounters.

Mobile vaccine equity enhancement program overview		
Total vaccine encounters		
Number of encounters for COVID vaccination with MVeeP	Number of patients	Percentage of Total
1	5,469	64.00%
2	2,639	30.88%
3	366	4.28%
4	44	0.51%
5	26	0.30%
6	1	0.01%
Total	8,545	100.00%
Homebound vaccine encounters		
1	456	76.90%
2	119	20.07%
3+	18	3.04%
Total	593	100.00%
Number of vaccine doses by age		
Patient age at time of vaccine	Number of vaccines administered	Percentage of total
0–4	45	0.37%
5–11	342	2.82%
12–19	871	7.19%
20–29	1,384	11.42%
30–39	1,634	13.49%
40–49	1,598	13.19%
50–59	2057	16.98%
60–69	2,134	17.61%
70–79	1,218	10.05%
80+	834	6.88%
Total	12,117	100.00%

In Worcester County, persons 65 years and older represent 17.3% of the population (19).

### Diversity, equity, and inclusion mission

The stated goal of the MVeeP was to enhance equity in vaccine delivery. Over the 24 months of operation, the program vaccinated a higher percentage of black and hispanic patients and a significant percentage of patients for whom English was not their primary language, indicating effectiveness of the MVeeP focus. These results are represented in Table 3.

**Table 3:** MveeP vaccine recipient race, ethnicity, and preferred language.

Vaccine administrations by patient reported ethnicity			
Ethnicity	Vaccine doses administered	Percentage of total	Worcester county data*
Asian	546	5%	5.6
Black/African American	1,276	11%	6.6
Hispanic	3,546	29%	12.8
White	5,000	41%	74.5**
Other and Unknown	1,749	14%	
	12,117	100%	

# Of vaccines administered by language spoken by patient	
Primary language spoken by patient	Number of vaccine doses (% of total)
English	9,015 (77)
Spanish	1,946 (17)
Portuguese	437 (4)
Vietnamese	104 (1)
Albanian	25 (0.2)
Arabic	80 (1)
Chinese (Mandarin)	1 (0)
Not reported	122 (1)

*Source: https://www.census.gov/quickfacts/worcestercountymassachusetts accessed on 3/17/23. ** “White alone” as reported by the US Census Bureau.

**Figure 2:**
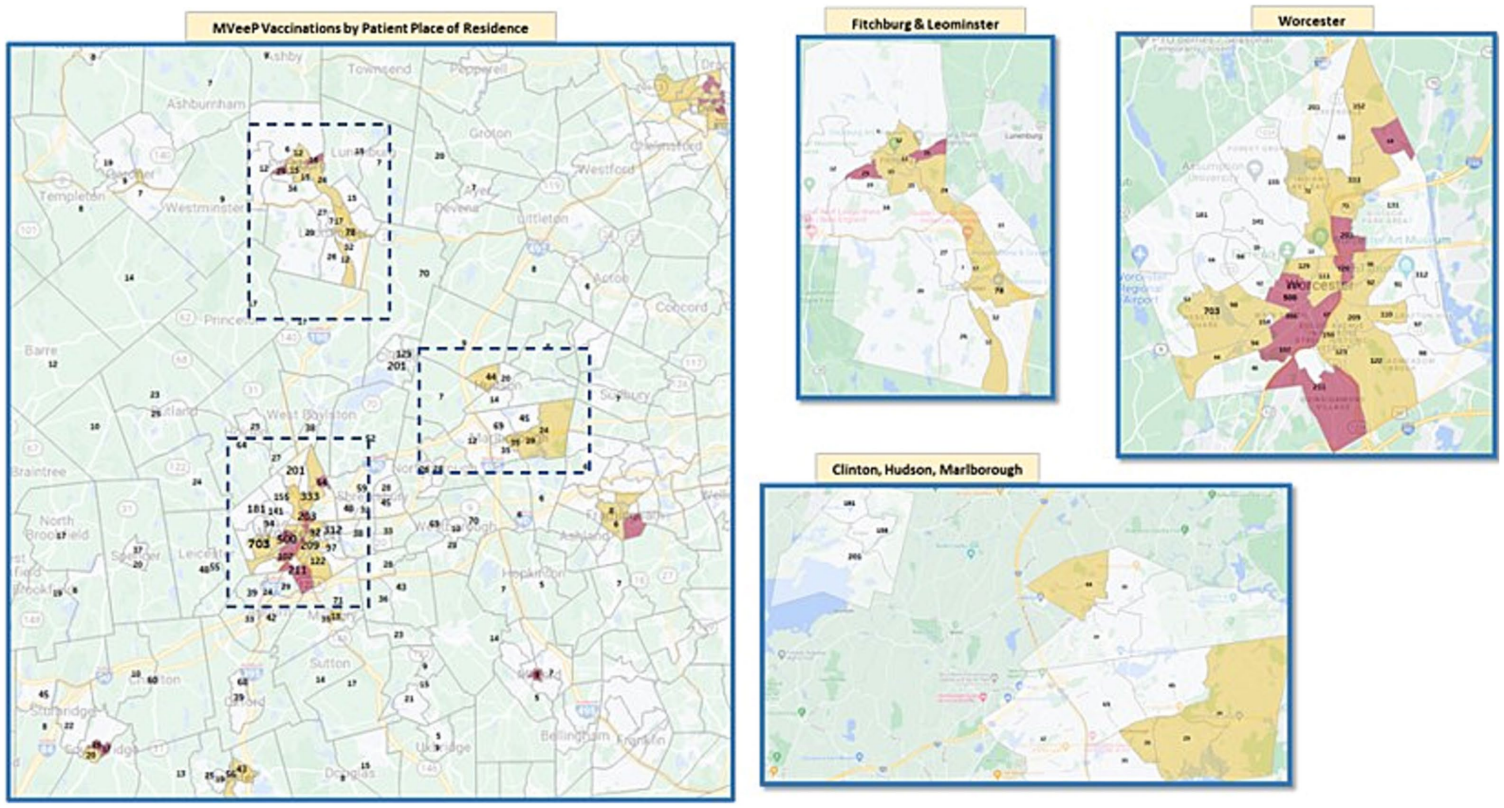
MveeP vaccinations delivery cross referenced with Social Vulnerability Index.

Figure 2 demonstrates the geographic locations of MVeeP clinics with overlaid census tract social determinants of health data. Census tracts are shaded in maroon if they have Social Vulnerability Indices (SVIs) in the 90th percentile and are therefore considered “most vulnerable” and shaded in beige if they have SVIs in the 75th to 89th percentiles (considered “very vulnerable”). The numbers on the map represent the number of patients who live in each tract who were served by our program.

### Site/partner selection

The MVeeP program was managed by the Office of Community Health Transformation & Community Benefits (CB) department of our Health System. The CB team used their contacts in the community to identify individuals who had challenges accessing vaccines. The team also identified suitable sites and times to facilitate easy patient access to clinics being held. MVeeP considered its service area to be the entire region of Massachusetts served by UMass Memorial Health (i.e., Massachusetts DPH Region 2). Sites were selected with as much lead time as possible, based on the following considerations: community interest, availability of vaccine, the State’s guidance for vaccine eligibility, consideration of maximal benefit among those with barriers to vaccines, and the factors of Social Determinants of Health (SDoH). Once sites were selected, MVeeP team members partnered with trusted community spokespeople to answer questions about vaccination and to provide information so that individuals were able to make an informed decision about vaccination. Specific foci for MVeeP were those with limited language proficiency, undocumented individuals, people of color, and those with mobility or technological barriers. Within this construct, five general types of events emerged: Community Site Events, Residential Site Events, Employment Events, Testing Site Events, and Mobile Events. Most vaccination events comprised more than one type of intervention and each are described below:

1. Community Site Events – Community events relied on centrally located community spaces such as town halls, places of worship, schools, etc. to host vaccination clinics that were publicized to a specific community. All events were open to the general public according to Massachusetts DPH mandate but focused publicity prior to each event helped to ensure that the maximum impact was directed at the community of interest.
2. Residential Events – Especially early in the vaccination program, much of the emphasis was understandably placed on older adult patients. In order to accommodate those that might have difficulty traveling from a senior living facility, especially those classified as senior affordable housing by the State of Massachusetts, special effort was made to work with these facilities in order to vaccinate their residents.
3. Employment Events – In a number of cases, the program engaged with employers to make vaccination clinics available on site at the place of work. This was a useful strategy not only because it allowed access to a large number of individuals and in certain worker categories that were high priority for vaccination but also because it allowed the program to address those who had long commutes and who could not afford to take time off of work to get vaccinated.
4. Testing Site Events – UMass Memorial Health also worked collaboratively with the State of Massachusetts to operate one of the Commonwealth’s Stop The Spread (STS) testing centers. This venue, located in downtown Worcester, Massachusetts, was another ideal venue for vaccination delivery.
5. Mobile Events – The MVeeP program recognized that the primary barrier for a number of patients who wanted to be vaccinated were mobility limitations and lack of access to transportation. For this reason, a priority of our program was to make vaccination available to homebound individuals and to those with mobility limitations in their homes. At each event, “strike teams” were used to perform vaccinations for patients who lived in a reasonable radius from the main location of the event. Prior to each event, a list of home-based visits was generated and sufficient two-person “strike teams” were created from among available MVeeP staff to complete these visits. The locations were plotted on a map and then the list of patients divided among the strike teams, roughly by sector and reasonable travel routes. This was accomplished prior to the event so that each team knew the addresses and patients that they were responsible for vaccinating. Just prior to departing, teams were supplied with adequate vaccines to accomplish their administrations while ensuring that all vaccines could be given within the allotted time since the vaccine vial was punctured.

Toward the end of many vaccine clinics, members of the MVeeP staff would also be dispatched to local neighborhoods to find individuals that qualified for vaccination. All necessary equipment was taken with them so that vaccinations could be delivered at the locations in which willing patients were found. All locations were used with the consent of their owners and included but were not limited to; barber shops, nail salons, homeless shelters, store fronts, city busses, private residences, and restaurants.

### Acquisition, transportation, cold chain, and distribution logistics

In order to ensure that sufficient vaccine was obtained prior to each event, members of the MVeeP team communicated the day before each event with the pharmacy staff at the Medical Center and requested a number of doses sufficient to complete the event. Confirmation was sent establishing that sufficient quantities would be made available. Each vaccine type used in this intervention (Moderna, Pfizer, and Johnson and Johnson) had different short term and long term storage requirements (20, 21). Long term cold chain storage requirements were maintained by the medical center pharmacy and, once thawed, short term requirements were maintained by the MVeeP team during vaccine events. On the morning of each vaccine event a member of the MVeeP team would go to the Medical Center and retrieve the vaccine doses contained in a specialized cooler that had been validated for 12 h of continuous temperature control within a certain range. Pick up time for each event was coordinated with the pharmacy on the day prior to the event. A continuous temperature reading was accomplished using a temperature probe that was monitored throughout each event by MVeeP staff. Because the vaccine was maintained at this temperature, it allowed unused vials to be returned to the pharmacy at the end of the event in the unlikely event that there were unexpectedly low numbers of patients at the community vaccine event. This ensured that no vials of vaccine were ever wasted or were out of temperature range and could not be administered. During the intervention, there were no unacceptable temperature excursions measured in the vaccine storage device and all unused vaccines were returned to the pharmacy in usable condition if not administered. Supplies required to administer the vaccines, including syringes, needles, and vaccine cards were supplied with the doses at the time of pick up. The pharmacy also supplies epi-pens and benadryl for use in the event of a vaccine reaction.

For each event, in addition to the vaccine doses themselves, significant amounts of supplies and infrastructure were required. As each event included between 30 and several hundred participants, items such as radios, tables, chairs, disposable medical supplies, IT equipment, hand sanitizer, etc. were required to be delivered to each site. This was accomplished using a dedicated 15-foot moving van and supply crew that traveled with the MVeeP program for its duration. These individuals were assigned from the Medical Center facilities staff and were permanent members of the MVeeP team. Before and after each event they assisted with delivery of supplies, unloading, set up, and retrieval of all logistical items needed to make the program successful.

### It infrastructure and mandatory reporting through MIIS

In order to ensure accurate tracking of patients who had received a vaccine, to ensure appropriate follow up if a vaccine reaction occurred, and to comply with the State of Massachusetts requirement that all vaccine administrations were logged into the MIIS system, a robust IT infrastructure was required to manage the MVeeP program. For this reason, MVeeP used the Epic Rover module in an innovative fashion in order to enable on site documentation of vaccine administration. To our knowledge, this was the first such use of Epic Rover in this fashion. Enabling this was a separate instance of the Epic EHR that was modified specifically for this use. Our Epic module communicated directly with the MIIS system, and therefore using it satisfied all documentation and reporting requirements for safe vaccine administration.

In addition to the EHR infrastructure, laptop computers and air cards were deployed with each MVeeP mission in order to ensure adequate connectivity. Air cards from multiple cellular carriers were used to ensure that in any given location adequate coverage could be achieved.

### Staffing–volunteers, EMS personnel, employed staff

Throughout the course of the pandemic, the State of Massachusetts adopted a progressive policy of allowing allied health disciplines authorization to administer vaccinations. Our program made special use of dentists, dental hygienists, EMT-Basics and EMT-Paramedics, physicians, and nurses. Because the MVeeP program was being rolled out simultaneously with other large vaccination efforts statewide, and because many health professionals were already deployed to front line and patient facing locations, volunteers (fully onboarded and background checked at the Medical Center) were used as vaccinators. All vaccinators received specialized training and were supervised by core members of the MVeeP staff. Finally, the City of Worcester, the home city of the UMass Memorial Medical Center, also collaborated on a number of vaccine events, supplying staffing and capabilities at these co-run events.

### Project management

Through its Center for Innovation and Transformational Change (CITC), project management support was provided to the MVeeP effort and was integral in the planning, resourcing, and execution of all aspects of the vaccination effort. This group also provided a critical link with the larger vaccination effort being undertaken by the Health System to ensure vaccine availability for all of its patients. Following Lean methodology, the CITC staff organized and streamlined the MVeeP effort to reduce waste and to increase efficiency of the effort.

### Interpreter services

Given the mission of MVeeP in providing more equitable access to COVID-19 vaccination, we worked closely with the UMass Memorial Medical Center Interpreter Services Department to ensure several countermeasures designed to provide adequate interpretation capability. First, vaccine information packets were created in English as well as the five other most commonly spoken languages in our area (Spanish, Portuguese, Albanian, Vietnamese, and Arabic). These included Emergency Use Authorization (EUA) material required for distribution with the vaccine from the Food and Drug Administration (FDA) as well as a number of other documents and resources created by our program that were professionally translated by the Medical Center Interpreter Services Department. Second, the Medical Center provided its interpreter hotline for both audio and video translators during all MVeeP events. In addition, as many of the MVeeP staff were employed by the UMass Memorial Medical Center Ronald McDonald Care Mobile, many had multiple language fluencies. During this intervention, based on the countermeasures above, we did not encounter any individuals that were not able to be consented and vaccinated safely due to language barriers.

### Medical support (hotline, on site and in home support)

As the program began, the MVeeP team recognized that many patients who would be served by this effort would not have easy access to medical follow-up or primary care physicians. This was due in part to the difficulty in accessing outpatient care during this time, that was itself a product of decreased staffing and office hours in the throes of the pandemic and partly due to the fact that many of our patients were undocumented or simply did not have well-established primary care. Recognizing this, the MVeeP team worked with local EMS agencies and the program leadership to ensure that not only were there physicians and Nurse Practitioners trained in emergency response on scene for each vaccination event but that a hotline number was provided to the patients as a part of each patient’s vaccine information packet. This hotline was staffed 24 h per day, 7 days per week by an Emergency Physician and was available for any patient who had questions regarding the vaccine or any side effects or symptoms following its administration.

### Adverse events

During the course of the entire first 12 months of the vaccine program, until vaccine scarcity was no longer a consideration, zero doses of COVID-19 vaccine were wasted or given to non-qualifying individuals. In fact, due to overfills of vaccine vials, many more vaccine doses were given out over the course of the program than would have been expected if the standard number of vaccines were drawn from each vial. In addition, no serious reactions occurred during the observation period after vaccine administration and during only two instances was Benadryl given for minor vaccine reactions. Zero patients required epinephrine.

## Discussion

The described vaccine effort was intended as an effort to enhance equity in vaccine delivery in a specific community of Central Massachusetts during the COVID-19 pandemic. This model provides several useful lessons learned for future pandemics that may require mass vaccination efforts, and also demonstrates some of the challenges inherent in providing a new type of vaccine to a population with myriad barriers to accessing it.

First, our program did demonstrate that mobile vaccination, even with vaccines that require specialized cold-chain logistics like the FDA approved vaccines, can be done safely. All vaccines were maintained within acceptable temperature ranges and no vaccine was wasted due to breakdown in cold-chain logistics. It is noteworthy that support from the medical center, which had access to extensive freezer capacity, allowed access to vaccine that was safely stored for longer periods of time prior to each event according to manufacturers guidelines. This was key to the success of the intervention. The program maintained strict adherence to vaccination guidelines and had no serious adverse events. Presence of on-site medical support and a medical hotline were important to ensuring that vaccines were delivered safely and that community members felt safe receiving the vaccine and supported thereafter.

Importantly, due to the logistical complexity of this vaccination program, and the challenges associated with doing it in a mobile fashion, a health center or other institutional partner is key to success. Our home institution supplied multiple infrastructural resources as described above in order to ensure safe and effective program administration. Clear direction from health system leaders is also required to marshal and maintain this support.

The existence of the Office of Community Health Transformation & Community Benefits, and specifically the staff of the UMass Memorial Medical Center Ronald McDonald Care Mobile as the home department for such an intervention, was incredibly important to the success of the initiative. Critical in this respect were the deep connections that this office and program had with community and city leaders. This was important not only as the program sought to select sites but also as it worked collaboratively with these leaders to encourage vaccinations and to allay fears that some in the community had expressed. In addition to the CB office, the Corporate Relations and Concierge Medicine Department of the UMass Memorial Health CarePath Program was critical in accessing employers that represented hundreds of employees in our region.

The role of the *Vaccine Navigator* was a crucial intervention that we feel played an outsized role in the success of our program. These staff members formed a vital link to the community and were able to contact patients in real time and to quickly ensure that all available slots on a given day were filled.

The program was especially well positioned to address vaccine distribution inequity and succeed in its goal of focusing on non-white and non-English speaking populations. African-American and Hispanic populations made up 11 and 29% of individuals who received vaccines through our program while only representing 6.6 and 12.8% of the population of Central Massachusetts, respectively (22). In addition, as demonstrated in Figure 2, our program was highly effective at providing access for patients who live in communities with high Social Vulnerability Indices. Other programs focused on mobile vaccine delivery have demonstrated success in vaccinating hard to reach populations. Given the importance of local specificity when it comes to targeting these communities, direct comparisons of success are difficult, but this intervention joins a small list of published literature detailed successful methods of ensuring equitable access to vaccination (9).

While no structured assessment of feedback such as survey or focus group was employed, the program received outstanding anecdotal feedback from our partners. Numerous community organizations expressed support for the approach and many requested multiple return visits and subsequent vaccine clinics due to the success of previous events. In addition, we were pleased that several thousand participants chose to receive second doses through our program despite expanding access through other sites throughout the period of intervention.

Direction of future investigation in this area should include standardizing metrics for success of mobile vaccine programs, and development of best practices for program administration. Although assessment of domains such as community engagement can be difficult to measure precisely, ensuring ways of ongoing assessment of success during a vaccine program are critically important. During future pandemics and smaller level vaccine-amenable disease outbreaks (e.g., Mpox) it will be vitally important to ensure equity in designing vaccine distribution.

### Acknowledgement of limitations

The program did experience difficulties, especially early on, with patient scheduling and the process of monitoring arrivals to ensure that all vaccine doses could be administered at each event. This was largely combated by the work of the *Vaccine Navigators* and program staff actively monitoring vaccine dose administration and remaining dose availability. In addition, by comparison to the larger mass vaccination sites in the State of Massachusetts and elsewhere, the scalability of our program was limited by the significant investment of time and resources in the administration of each vaccine dose. While this meant that the program was less efficient on a strictly dollar-per-dose basis, it is also acknowledged that many of the patients vaccinated by this program had significant difficulty accessing mass vaccination sites and many patients noted that the MVeeP program was their only opportunity to receive the vaccine. Many of these patients were either at very high risk of severe COVID-19 infection by virtue of age or comorbidity (i.e., the older adult and multiply comorbid patients with COVID-19 risk factors) or were members of groups that experienced higher than expected morbidity and mortality during the early phases of the COVID-19 pandemic – especially among communities of color. On limitation in terms of data collection in this intervention was that no specific data was recorded related to the percentage of patients with mobility challenges and their use of the service. Although this is captured to some extent in the number of home-bound visits that were performed, a more specific accounting of these patients and specific challenges involved would improve future interventions.

Despite significant effort to reach all communities it is acknowledged that some individuals in our service area were likely missed. Our approach depended upon engagement from community organizations and individuals without connections to these partners may have been less likely to receive notification of our events and to access the vaccine. The team attempted to mitigate this by incorporate a system of on-site registration to allow neighborhood residents to be vaccinated even if they had not been aware of the event prior. We used social media, communications such as newsletters and fliers, and word of mouth advertising to make as many people aware of the process as possible. However, future work should focus on the best ways to ensure maximum engagement, especially among isolated community members to ensure the greatest equity possible in terms of access.

In conclusion, the Mobile Vaccine Equity Enhancement Program (MVeeP) demonstrates one successful model for meaningful community engagement and the deployment of medical countermeasures in the midst of a global pandemic in a way that was specifically designed to address equity.

## Data availability statement

The raw data supporting the conclusions of this article will be made available by the authors, without undue reservation.

## Ethics statement

The studies involving humans were approved by UMass Chan Medical School Human Subjects Institutional Review Board. The studies were conducted in accordance with the local legislation and institutional requirements. Written informed consent for participation was not required from the participants or the participants’ legal guardians/next of kin in accordance with the national legislation and institutional requirements.

## Author contributions

JB: Conceptualization, Project administration, Resources, Supervision, Writing – original draft, Writing – review & editing. OB: Conceptualization, Data curation, Formal analysis, Resources, Writing – original draft, Project administration, Supervision, Writing – review & editing. CaiM: Methodology, Project administration, Supervision, Writing – review & editing. JF: Investigation, Resources, Supervision, Writing – review & editing. PL: Resources, Methodology, Supervision, Writing – review & editing. NS: Project administration, Validation, Supervision, Writing – review & editing. JI: Project administration, Resources, Validation, Supervision, Writing – review & editing. RK: Methodology, Supervision, Writing – review & editing. ST: Conceptualization, Supervision, Writing – review & editing. AH: Visualization, Writing – original draft, Methodology, Supervision, Writing – review & editing. PM: Methodology, Resources, Supervision, Writing – review & editing. RB: Data curation, Methodology, Project administration, Writing – review & editing. GD: Supervision, Resources, Writing – review & editing. MG: Methodology, Resources, Writing – review & editing. CC: Resources, Supervision, Methodology, Writing – review & editing. JS: Methodology, Data curation, Formal analysis, Visualization, Resources, Supervision, Writing – review & editing. KE: Supervision, Conceptualization, Methodology, Resources, Writing – review & editing. NV: Project administration, Resources, Writing – review & editing. SH: Resources, Supervision, Writing – review & editing. KF: Writing – review & editing, Project administration, Supervision. RG: Resources, Writing – review & editing, Supervision. TH: Resources, Writing – review & editing, Methodology. VM: Project administration, Supervision, Writing – review & editing. TV: Writing – review & editing, Formal analysis, Software, Validation, Visualization. MB: Resources, Writing – review & editing, Project administration. TJ: Resources, Writing – review & editing, Software. CatM: Software, Writing – review & editing, Resources. WS: Project administration, Resources, Writing – review & editing. IP: Resources, Writing – review & editing, Project administration, Methodology. MR: Resources, Writing – review & editing, Project administration. CL: Project administration, Resources, Writing – review & editing. ML: Project administration, Resources, Writing – review & editing. ER: Project administration, Resources, Supervision, Writing – review & editing. LG: Project administration, Resources, Writing – review & editing, Methodology. AG: Project administration, Resources, Writing – review & editing, Supervision. SB: Project administration, Resources, Supervision, Writing – review & editing. ED: Project administration, Resources, Supervision, Writing – review & editing. MM: Project administration, Resources, Supervision, Writing – review & editing.
